# Monte Carlo modeling of a ^60^Co MRI-guided radiotherapy system on Geant4 and experimental verification of dose calculation under a magnetic field of 0.35 T

**DOI:** 10.1093/jrr/rry087

**Published:** 2018-11-08

**Authors:** Hiroyuki Okamoto, Shie Nishioka, Kotaro Iijima, Satoshi Nakamura, Tatsuya Sakasai, Yuki Miura, Mihiro Takemori, Hiroki Nakayama, Yuichiro Morishita, Morihito Shimizu, Yoshihisa Abe, Hiroshi Igaki, Yuko Nakayama, Jun Itami

**Affiliations:** 1 Department of Radiation Oncology, National Cancer Center Hospital, 5-1-1 Tsukiji, Chuo-ku, Tokyo, Japan; 2 Department of Radiological Sciences, Graduate School of Human Health Sciences, 7-2-10 Higashi-Ogu, Arakawa-ku, Tokyo, Japan; 3 National Metrology Institute of Japan, 1-1-1 Umezono, Tsukuba, Ibaraki, Japan

**Keywords:** MRI, Monte Carlo, MRI-guided, modeling

## Abstract

Our purpose was to establish the commissioning procedure of Monte Carlo modeling on a magnetic resonance imaging–guided radiotherapy system (MRIdian, Viewray Inc.) under a magnetic field of 0.345 T through experimental measurements. To do this, we sought (i) to assess the depth–dose and lateral profiles generated by the Geant4 using either EBT3 film or the BJR-25 data; (ii) to assess the calculation accuracy under a magnetic field of 0.345 T. The radius of the electron trajectory caused by the electron return effect (ERE) in a vacuum was obtained both by the Geant4 and the theoretical methods. The surface dose on the phantom was calculated and compared with that obtained from the film measurements. The dose distribution in a phantom having two air gaps was calculated and measured with EBT 3 film. (i) The difference of depth–dose profile generated by the Geant4 from the BJR-25 data was 0.0 ± 0.8% and 0.3 ± 1.5% for field sizes of 4.5 and 27.3 cm^2^, respectively. Lateral dose profiles generated by Geant4 agreed well with those generated from the EBT3 film data. (ii) The radius of the electron trajectory generated by Geant4 agreed well with the theoretical values. A maximum of ~50% reduction of the surface dose under a magnetic field of 0.345 T was observed due to elimination of the electron contamination caused by the magnetic field, as determined by both the film measurements and the Geant4. Changes in the dose distributions in the air gaps caused by the ERE were observed on the Geant4 and in the film measurements. Gamma analysis (3%/3 mm) showed a pass rate of 95.1%. Commissioning procedures for the MRI-guided radiotherapy system on the Geant4 were established, and we concluded that the Geant4 had provided high calculation accuracy under a magnetic field of 0.345 T.

## INTRODUCTION

Magnetic resonance imaging (MRI)-guided radiotherapy combined with innovative technologies offers new options for high-precision radiotherapy. Implementing MRI into radiotherapy can provide many advantages. MRI has high contrast in soft tissue, which enables a physician to more accurately determine target and critical structure volumes, compared with when using CT images. In addition, by using MR images instead of CT images, patients receive no radiation dose during the set-up before daily treatments.

**Fig. 1.**
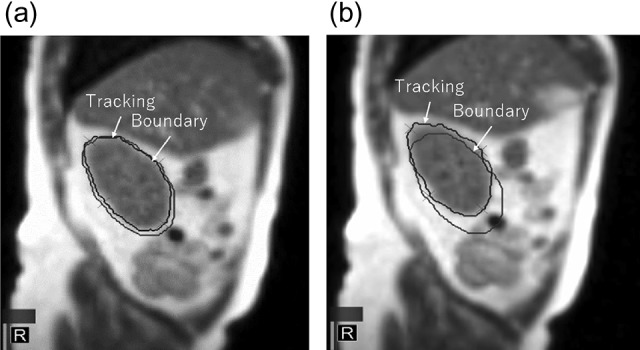
Tracking and boundary region in Cine-MR image. The tracking volume is (a) within the boundary (beam-on) and (b) beyond the boundary (beam-off).

Since 2015, clinical use of a commercial MRI-guided radiotherapy system, the MRIdian (ViewRay Inc., Cleveland, OH, USA) has been reported, and treatment sites have included the head and neck, thorax, abdomen and pelvis [1–6]. This system allows margin reduction by using real-time MR images. The MRIdian has a horizontal solenoidal superconducting superior–inferior magnetic field of 0.345 T, offers whole-body MRI, and employs three ^60^Cobalt sources 120 degrees apart mounted on a ring gantry, allowing MRI-guided radiotherapy using 3D conformal radiotherapy and step-and-shoot intensity-modulated radiotherapy (IMRT) [7]. Notably, gated radiotherapy is possible based on the boundaries on real-time MR images (Cine), as shown in Fig. 1, and this treatment principle can lead to a reduction of the irradiated region by reducing geometrical intrafraction uncertainty. In addition, MRIdian has a sophisticated integrated system and an established operability, permitting fast and efficient on-line adaptive radiotherapy [5, 6].

Several studies have reported dosimetric changes due to changing secondary electron trajectories by Lorentz force under the presence of a magnetic field, called the ‘electron return effect (ERE)’ [8–15]. Especially at the tissue–air boundaries, a large dosimetric impact may be induced due to the ERE, and the Monte Carlo study has been undertaken to clarify dose deposition phenomena under various conditions, including low- and high-strength magnetic fields. However, procedures for implementing Monte Carlo into such analyses have not been reported for ^60^Co MRIdian. In addition, a reduction of surface dose could be expected by elimination of electron contamination by the magnetic field, but the quantitative changes remain unclear. Additionally, the dose calculation accuracy for the surface dose should be verified in a Monte Carlo simulation by comparison with experimental measurements.

Our objective in this study was to provide helpful information for implementing and commissioning Monte Carlo modeling for ^60^Co MRIdian through experimental verifications, which can then be applied widely to allow for ERE in commercial MRI-guided radiotherapy systems in the future.

## MATERIALS AND METHODS

### Monte Carlo modeling

**Fig. 2.**
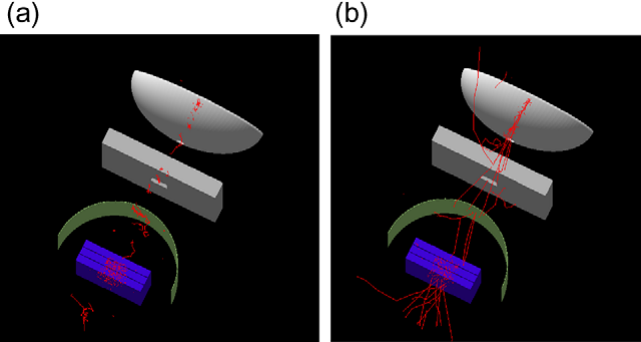
Modeled ^60^Co MRIdian in Geant4 (a) with and (b) without a magnetic field of 0.345 T. Only electron trajectories are shown.

Monte Carlo modeling for ^60^Co MRIdian was conducted using the software toolkit Geant4 (Ver. 10.2 patch02) [16, 17]. As shown in Fig. 2, the major components in the treatment head modeled in Geant4 are the cylindrical shape of a ^60^Co source with a diameter of 2 cm and a height of 2.7 cm, a double-focused multileaf collimator (MLC), and the inner surface of the bore wall. The density of ^60^Co was assumed to be uniform inside the source. ViewRay MLC has a tongue-and-groove design on both sides and the tip of the leaf, to reduce radiation leakage, because a collimator jaw is not equipped. The double-focused ViewRay MLCs can move on the arc so that the MLC faces at its tip stay parallel to the beam path and the penumbra does not depend on MLC positions. Because the ViewRay MLCs have complicated structures and movements, the MLC modeling was simplified in this study: In the Geant4 simulation, the tongue-and-groove design was not modeled, and the MLC movement was perpendicular to a beam axis with equivalent beam taper for definition of field sizes. The wall of the bore is made of glass fiber, and general material components (SiO_2_ = 53%, Al_2_O_3_ = 15%, CaO = 21%, MgO = 3%, B_2_O_3_ = 8%) [18] were used because the specific composition could not be clarified. The static magnetic field of 0.345 T was simulated in the superior–inferior direction using G4UniformMagField class in the Geant4 code. The Geant4 provides many user-selectable parameters for calculations. In this analysis, standard electromagnetic physics [19] was used, with a cut-off range of 10 μm for calculating the surface dose and 1 mm for other calculation conditions, as mentioned later.

### Validation of modeling

Validation of the modeling accuracy was conducted by the following procedures.

#### Test I: depth–dose and lateral dose profile

Test I was to assess beam quality by comparison of the depth–dose and the lateral dose profiles between Geant4 and the measurements or the published ^60^Co data for field sizes of 4.2 and 27.3 cm^2^. The calculated depth–doses were compared with the published ^60^Co data from *British Journal of Radiology* Supplement 25 (BJR-25) [20], because there was no commercial water tank with a 3D scanning system compatible with a magnetic field in the commissioning, and we could not measure the depth–dose profile in MRIdian. Alternatively, spot-checks of tissue-maximum ratio (*TMR*) at depths of 5 and 10 cm for field sizes of 2.1 to 27.3 cm^2^, compared with BJR-25, were performed using a developed 1D water tank compatible with a magnetic field (Taisei Medical, Osaka, Japan). A MR-compatible chamber (A1SL, Standard Imaging, Inc. WI, USA) was used for this measurement under the presence of the magnetic field. The differences between them were within ~2%, as shown in Table 1. Lateral dose profiles for field sizes of 4.2 and 27.3 cm^2^ were measured with Gafchromic^TM^ EBT3 film (Ashland Inc., NJ, USA) under the presence of the magnetic field. The EBT3 film was placed at a 5 cm depth with a source–surface distance (SSD) of 100 cm, and the irradiated film was then calibrated to convert the net optical density to the absorbed dose in water using the dose–response curve obtained in MRIdian.

**Table 1.** *TMR* with source–chamber distance of 105 cm from MRIdian and BJR-25

Field size (cm^2^)	2.1	4.2	6.3	10.5	21.0	27.3
5 cm depth						
MRIdian	0.797	0.824	0.849	0.874	0.902	0.908
BJR-25 [20]	0.783	0.821	0.848	0.877	0.904	0.910
Difference (%)	1.8	0.3	0.1	−0.3	−0.2	−0.3
10 cm depth						
MRIdian	0.582	0.611	0.645	0.694	0.752	0.767
BJR-25 [20]	0.575	0.614	0.649	0.699	0.752	0.769
Difference (%)	1.1	−0.6	−0.6	−0.7	0.1	−0.3

Tissue-maximum ratio (*TMR*), MRIdian = magnetic resonance imaging–guided radiotherapy system (Viewray Inc.), BJR-25 = *British Journal of Radiology* Supplement 25 data.

#### Test II: Three tests to assess the calculation accuracy of Geant4 in the presence of a magnetic field

**Fig. 3.**
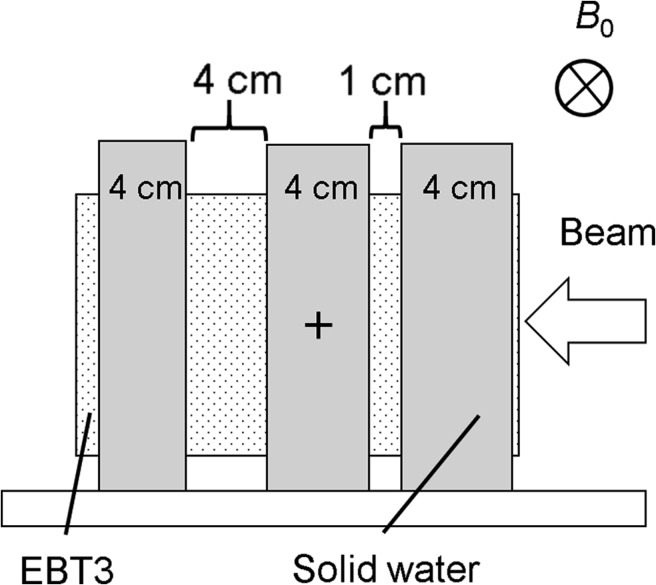
Measurements of dose distribution in a solid water phantom with two air gaps (thicknesses of 1 and 4 cm) with EBT3 film to assess the dosimetric changes in the air gaps caused by ERE. *B*_0_ = static magnetic field.

- The electron trajectory radii R for various magnetic fields and electron energies in the presence of the magnetic field were evaluated using scoring particle positions in sensitive volumes with a grid size of 0.1 mm in a Geant4 simulation, and the theoretical values were also obtained using the following equation:
  (1) R=p/(qB)
  where *R* is the radius of electron trajectory and *p* is the momentum of the electron, which can be derived from the equation $E^{2}={\left(m_{0}c^{2}\right)}^{2}+{\left(cp\right)}^{2}$ (*E*: total energy, *m*_0_: electron rest mass). The symbols *q* and *B* are the electron charge and the magnetic field strength, respectively. We estimated the uncertainty to be 0.1 mm from the calculation grid size in this simulation.
- Surface doses on a solid water phantom (Sun Nuclear corporation, Melbourne, FL, USA) were calculated and compared with film measurements for field sizes of 4.2–21.0 cm^2^, because electron contamination from the inner surface of the bore can be eliminated by the Lorentz force, and scattered electrons move along the superior–inferior static magnetic field. In the Geant4 simulation, 28 μm of sensitive volume, as the active layer of the EBT3 film [21], was modeled on the surface of the water to calculate the energy deposited with and without a magnetic field of 0.345 T. In the film measurements, films were placed at the surface and at 5 cm depth in the solid water phantom with a SSD of 100 cm. The surface doses were obtained as relative doses normalized to the film dose at 5 cm depth. During installation of the MRIdian, we had the opportunity to shut down the magnetic field of 0.345 T, and the surface dose could be measured with the EBT3 film with and without the magnetic field.
- As shown in Fig. 3, the dose distribution in a solid water phantom with two air gaps (1 and 4 cm) was calculated for a field size of 4.2 cm^2^, and the dosimetric changes in the air gaps caused by ERE were assessed by comparison with the film measurements. To improve the dose calculation accuracy, the EBT film was also modeled in the phantom. A 3%/3 mm distance-to-agreement (DTA) with a threshold of 10% in gamma analysis [22] was employed for this test using the film analysis software DD-system (Ver. 10.21, R-TECH, Tokyo, Japan).

## RESULTS

### Test I: depth–dose and lateral dose profile

**Fig. 4.**
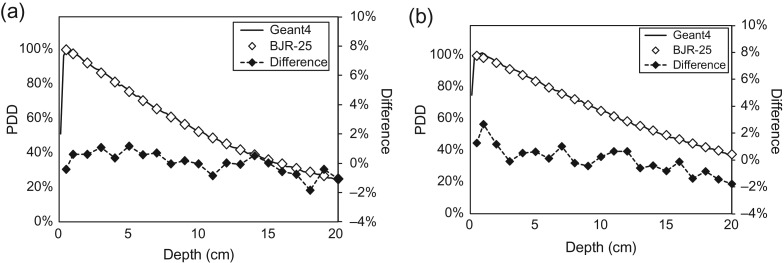
Depth–dose profiles from Geant4 and BJR Supplement 25 data for field sizes of (a) 4.2 and (b) 27.3 cm^2^.

**Fig. 5.**
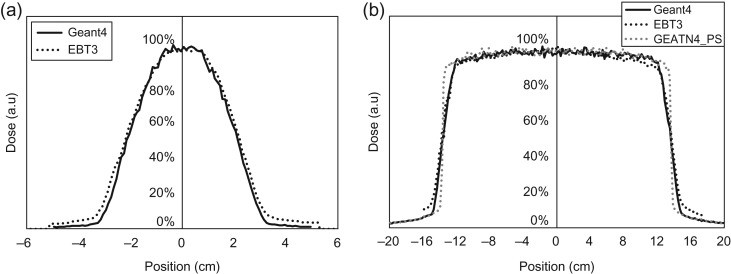
Lateral dose profiles for field sizes of (a) 4.2 and (b) 27.3 cm^2^, calculated by Geant4 and measured with EBT3 film. The gray dotted line represents the calculated dose profile using a point ^60^Co source.

Figure 4 shows the depth–dose profiles with a SSD of 100 cm from Geant4 and the BJR-25 data for field sizes of 4.2 and 27.3 cm^2^. The comparison between them shows 0.0 ± 0.8% and 0.3 ± 1.5% in a range of 0–20 cm depth for field sizes of 4.2 and 27.3 cm^2^, respectively. Figure 5 shows the lateral dose profiles for field sizes of 4.2 and 27.3 cm^2^, calculated by Geant4 and measured with the EBT3 film. The penumbra regions for doses of 20–80% for the field size of 4.2 cm^2^ in Geant4 and the EBT3 film were 1.28 and 1.31 cm, respectively, and 1.63 and 1.93 cm, respectively, for the field size of 27.3 cm^2^. The penumbra discrepancy for large field sizes might be caused from the aforementioned limitations of MLC modeling.

### Test II: calculation accuracy under a magnetic field

Table 2 shows the radii of electron trajectories in a vacuum for 1–6 MeV of electron energy at 0.3, 1.0 and 1.5 T in Geant4 and the theoretical values using Eq. (1). The Geant4 results agreed well with the theoretical values.

**Table 2.** Radii of electron trajectories in a vacuum for 1–6 MeV electron energy under 0.3, 1.0 and 1.5 T in Geant4 and the theoretical values

*E* (MeV)	1.0	1.0	1.0	3.0	6.0
*B* (T)	0.3	1.0	1.5	1.0	1.0
Geant4 (cm)	1.58	0.47	0.32	1.16	2.17
Theoretical value (cm)	1.58	0.47	0.32	1.16	2.16
Difference (cm)	0.0	0.0	0.0	0.0	0.0

*E* = electron energy, *B* = magnetic field strength.

**Fig. 6.**
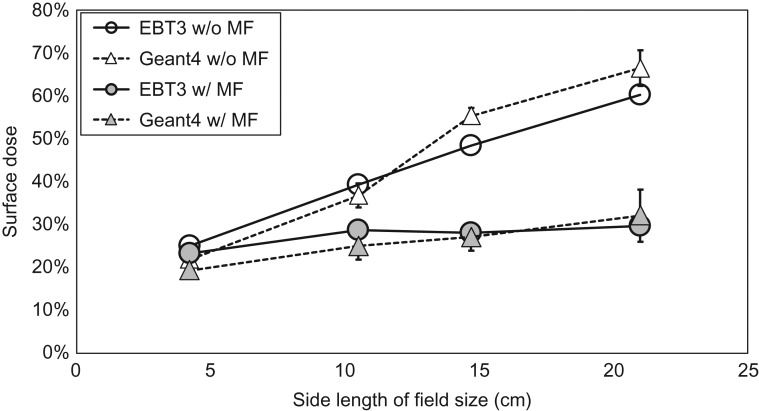
Surface dose with or without magnetic field, calculated by Geant4 and measured with EBT film.

Figure 6 shows surface doses with or without a magnetic field of 0.345 T, calculated by Geant4 and measured with the EBT film for field sizes of 4.2–21.0 cm^2^. The error bars in the film measurements were obtained from a standard deviation of film doses in the region of interest, and they are estimated to be ~1%. For the overall uncertainty in dose measured by the EBT3 film, Marroquin reported that it was 3.2% [23]. The relative doses in the vertical axis were normalized to the film dose at 5 cm depth. From the results, the surface dose increased with increasing field size, both with and without a magnetic field, and the Geant4 results agreed well with the film measurements. In addition, the surface doses for all field sizes with the magnetic field were lower than those without the magnetic field. The reduction ratios in the EBT film for field sizes of 4.2, 10.5, 14.7 and 21.0 cm^2^ were −7, −27, −42 and −51%, respectively. The corresponding reductions by Geant4 were −12, −32, −51 and −52%, respectively.

**Fig. 7.**
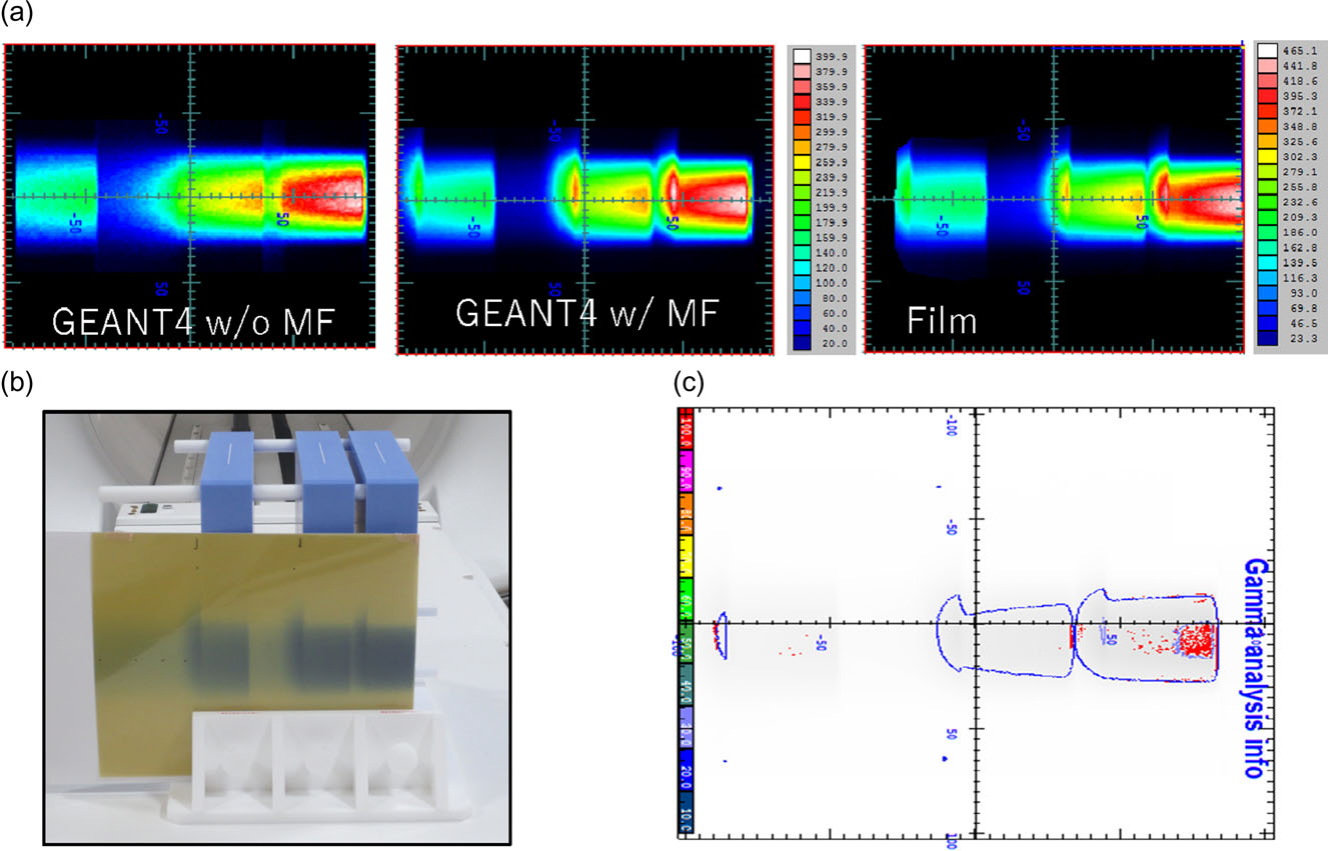
EBT film measurement for depth–dose with two air gaps in (a) the Geant4 without and with magnetic field, and the EBT3 film (unit of cGy); (b) the EBT3 film; and (c) Gamma analysis with a criterion of 3%/3 mm DTA (threshold = 10%). The red distributions represent the area that does not meet the criterion.

**Fig. 8.**
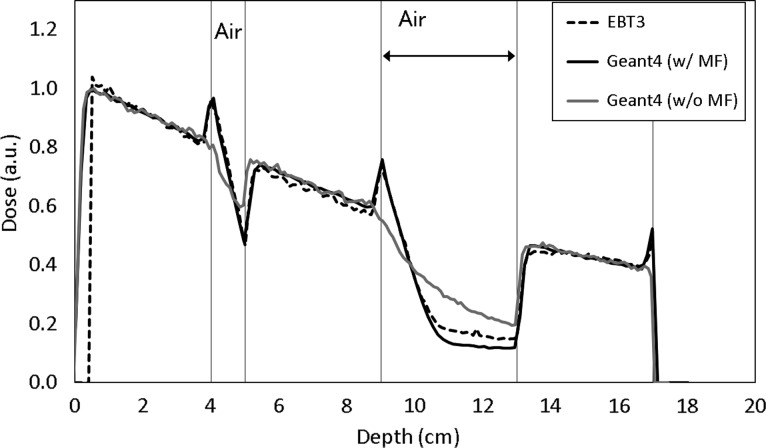
The measured depth–dose profile with the EBT3 film (dashed line) and the calculations by the Geant4 with magnetic field (‘G4 w/MF’, solid line) and Geant4 without magnetic field (‘G4 w/o MF’, gray line) in a water-equivalent phantom with two air gaps under the magnetic field. SW = solid water.

Figure 7 shows the depth–dose profile in the solid water phantom with two gaps (thickness of 1 and 4 cm). Changes in the dose distributions in the two air gaps due to ERE were observed both in the Geant4 results and the film measurements. Gamma analysis with a criterion of 3%/3mm indicated a pass rate of 95.1% with reference to the film measurements. The red distributions represent the area that does not meet the criterion (Fig. 7c). Figure 8 shows the measured depth-dose profile with the EBT3 film in the water-equivalent phantom with two air gaps under the presence of a magnetic field. It was observed that there were high and cold doses at the interface of the medium due to ERE in both the EBT3 film and the Geant4, such dose changes were also reported [9, 14].

## DISCUSSION

As mentioned above, because there is no commercial water tank compatible with a magnetic field, we could not obtain the depth–dose profiles in MRIdian. Use of the BJR-25 data was justified by comparison for spot-checking of *TMR* with the BJR-25 data. The differences fell within ~1% for almost all conditions. Similar results were reported by Park *et al.* [24]. The shapes of both the Geant4 results and the measurements were similar for the depth–dose profiles. The mean differences of 0.0% and 0.3% for field sizes of 4.2 and 27.3 cm^2^ were close to zero, and systematic deviation was not observed. The chamber response was changed under the presence of a magnetic field, as the function of the chamber response indicates the smallest changes [25]. However, while the magnetic field was perpendicular to the photon beam, it indicate the smallest changes with the direction of the chamber parallel to the that of the static magnetic field. In these *TMR* measurements, the chamber response changes could be ignored because the relative reading chamber was used, and the direction of the chamber was the same as that of the magnetic field.

For lateral dose profiles (Fig. 5), comparisons between the Geant4 results and the measurements showed good agreement. It was notable that the lateral dose profile had a greater penumbra region in MRIdian, compared with the typical penumbra of ~5 mm in the conventional linac. That was thought to be because MRIdian uses the comparatively larger size of a ^60^Co source to achieve the high dose rate of ~6 Gy/min at the isocenter, and this size is greater than the beam size of ~0.3 cm in the conventional linac [26]. For instance, Fig. 5b shows the lateral dose-profiles based on the assumption that the shape of the ^60^Co source in the MRIdian could be defined as a point (dotted line in gray color). The penumbra of this profile was 0.3 cm, comparable with that of the conventional linac.

Table 2 shows that the Geant4 results agreed well with the theoretical values. The magnetic field strength (*B*_0_) of 0.3 and electron energy (*E*) of 1 MeV resembled those of the MRIdian, and the radius of the electron trajectory was ~1.5 cm.

As shown in Fig. 6, surface doses for all field sizes with the magnetic field were lower than those without the magnetic field due to the aforementioned elimination of electron contamination. The reduction ratios were greater for larger field sizes; the irradiation area increases with a larger field size, and scattered electrons, which result in a greater contribution to the surface dose without a magnetic field, move along the superior–inferior static magnetic field. In contrast, electron streams generated by the magnetic field contribute to the out-of-field dose. For instance, it was reported that the patient’s jaw, ipsilateral shoulder and arm received unwanted doses during breast treatment [27].

There are various designs for integrating an MRI scanner with a treatment system [28]. Some machines use a magnetic field parallel to the beam central axis. In this case, the scattered electrons travel along the magnetic field, which results in an increased surface dose [29, 30].

As shown in Fig. 7, distortion of the dose distribution in the air region was observed, and hot spots were created at tissue–air interfaces. In clinical practice, air cavities in soft tissue, such as rectal gas and air passages, and low-density tissues such as the lung, etc. should be carefully considered to account for dosimetric changes, especially when using high-energy photon beams and strong magnetic fields [9, 31–33].

Magnetic fields can affect radiation measurement in several ways. As mentioned above, changing the electron trajectory by a magnetic field can change the chamber response to different beam angles [25]. Small gaps can occur around the chamber in a water-equivalent phantom, resulting in a few percentage points of change to the charge of the chamber [34]. Magnetic fields also affect the crystal orientation and polymerization of the active layer of radiochromic film [35, 36]. Many factors remain to be clarified regarding the impact of magnetic fields.

## CONCLUSION

Commissioning procedures for Monte Carlo modeling of a ^60^Co MR-guided radiotherapy system were established based on experimental verifications, and the high calculation accuracy of Geant4 under a magnetic field was demonstrated.
